# Epidemiologic Evidence on the Health Effects of Perfluorooctanoic Acid (PFOA)

**DOI:** 10.1289/ehp.0901827

**Published:** 2010-04-27

**Authors:** Kyle Steenland, Tony Fletcher, David A. Savitz

**Affiliations:** 1 Rollins School of Public Health, Emory University, Atlanta, Georgia, USA; 2 London School of Hygiene and Tropical Medicine, London, United Kingdom; 3 Mount Sinai School of Medicine, New York, New York, USA

**Keywords:** epidemiology, health effects, PFOA

## Abstract

**Objective and sources:**

We reviewed the epidemiologic literature for PFOA.

**Data synthesis:**

Perfluorooctanoic acid (PFOA) does not occur naturally but is present in the serum of most residents of industrialized countries (U.S. median, 4 ng/mL). Drinking water is the primary route of exposure in some populations, but exposure sources are not well understood. PFOA has been used to manufacture such products as Gore-Tex and Teflon. PFOA does not break down in the environment; the human half-life is estimated at about 3 years. PFOA is not metabolized in the body; it is not lipophilic. PFOA is not directly genotoxic; animal data indicate that it can cause several types of tumors and neonatal death and may have toxic effects on the immune, liver, and endocrine systems. Data on the human health effects of PFOA are sparse. There is relatively consistent evidence of modest positive associations with cholesterol and uric acid, although the magnitude of the cholesterol effect is inconsistent across different exposure levels. There is some but much less consistent evidence of a modest positive correlation with liver enzymes. Most findings come from cross-sectional studies, limiting conclusions. Two occupational cohort studies do not provide consistent evidence for chronic disease; both are limited by sample size and reliance on mortality data. Reproductive data have increased recently but are inconsistent, and any observed adverse effects are modest.

**Conclusions:**

Epidemiologic evidence remains limited, and to date data are insufficient to draw firm conclusions regarding the role of PFOA for any of the diseases of concern.

Perfluorooctanoic acid (PFOA, or C8) is a perfluorinated compound (PFC) that does not occur naturally but was introduced in the environment after World War II. PFCs are fluorocarbons with at least one additional atom or functional group; PFOA has a carboxylic acid group (C_7_F_15_COOH). Most people tested in the United States have PFOA in their serum (measured via perfluorooctanoate, the disassociated ion of PFOA), with a median of 4 ng/mL, but the source of exposure is not clear (Calafat et al. 2007b). PFOA has also been detected in the serum of populations in a number of industrialized countries (Fromme et al. 2009; Kannan et al. 2004; Lau et al. 2007). Limited data suggest that PFOA in water systems may result from wastewater treatment plants, which concentrate PFOA (Becker et al. 2008; Pistocchio and Loos 2009). Levels of PFOA in the serum appear to be decreasing in the U.S. population, because some large users have curtailed emissions (Calafat et al. 2007b; Olsen et al. 2007b).

Determinants of PFOA in the general population are known to include age (higher at younger and older ages), sex (males higher), and race (whites higher) (Calafat et al. 2007a, 2007b; Emmett et al. 2006a; Steenland et al. 2009a). However, none of these attributes are very strong predictors. For those living near a point source of contamination, contamination of drinking water has been identified as the major route of exposure (Emmett et al. 2006a; Hölzer et al. 2008; Steenland et al. 2009a).

PFOA has been used in the production of a wide variety of industrial and consumer products, such as Gore-Tex and Teflon. PFOA does not break down once in the environment, because of the strong carbon–fluorine bonds, leading to widespread buildup and bioaccumulation. Two studies provide estimates of the half-life for PFOA in humans. In a study of 26 retired fluorochemical production workers (mean years retired at initial blood collection, 2.6), followed up for 5 years, the median half-life was 3.4 years (Olsen et al. 2007a). In a study of 200 people who had been exposed via public water supplies and followed for 1 year after installation of filtration for the water supplies, the median half-life was 2.3 years (Bartell et al. 2010). The latter estimate suffers from the short follow-up time (although this population will be followed for 5 years), whereas the former suffers from small numbers and a lack of data at the time when exposure ceased.

PFOA is not metabolized in the body; its tissue distribution in humans is unknown, but studies in rats suggest it is likely to be present primarily in the liver, kidney, and blood (Kennedy et al. 2004; Lau et al. 2007). It is easily absorbed via the gastrointestinal tract in rats. It is not lipophilic, unlike chlorinated hydrocarbons, but binds to serum albumin and is excreted primarily from the kidney. It is not directly genotoxic (Andersen et al. 2008).

There is a considerable amount of animal data on the health effects of PFOA, which has been summarized by others [Kennedy et al. 2004; Lau et al. 2007; U.S. Environmental Protection Agency (EPA) 2005]. The relevance of animal (primarily rodent) data for humans is controversial because of a much shorter half-life in rodents (measured in days) and the possible dependence of some animal toxicity on a peroxisome proliferation mechanism that is likely to be less important in humans (see below).

PFOA induces tumors of the testicles, liver, and pancreas in rodents (Biegel et al. 2001; Sibinski 1987; U.S. EPA 2005) via dietary intake, and there is some evidence it also increases mammary tumors (Sibinski 1987; U.S. EPA 2005). The lowest doses at which effects have been observed in these rodent studies appear to be several orders of magnitude higher than human doses from drinking water contaminated at a level of 1 ng/mL.

In its draft risk assessment, the U.S. EPA (2005) concluded that evidence was suggestive that PFOA is carcinogenic in humans. In its review of that risk assessment, three of the four members of the EPA scientific advisory board concluded more strongly that PFOA was “likely to be carcinogenic in humans” (U.S. EPA 2006).

PFOA also reduces birth weight in mice and causes neonatal death in rats (Lau et al. 2007; U.S. EPA 2005). In mice it decreases the B-cell and T-cell immune responses, and in rats it results in atrophy of the spleen and thymus, causes hepatomegaly, and decreases levels of cholesterol (Kennedy et al. 2004; Lau et al. 2007; Loveless et al. 2006). Several experimental studies have reported that PFCs impair thyroid hormone homeostasis. Depression of serum triiodothyronine (T_3_) and/or thyroxine (T_4_) in PFOA- and PFOS-exposed rats and monkeys has been reported (Butenhoff et al. 2002; Lau et al. 2007), but without an expected corresponding elevation of thyroid-stimulating hormone (TSH) through feedback stimulation of the hypothalamic–pituitary–thyroid axis. However, it has been hypothesized these associations could be an artifact of the use of analog methods for assessment of free thyroid hormones and are absent if the equilibrium dialysis method is used (Chang et al. 2007; Lau et al. 2007).

One mechanism that has been proposed for these effects in rodents is peroxisome proliferation. In animals, PFOA is a strong peroxisome proliferator in the liver, and this proliferation has been shown to alter lipids, liver enzymes, and liver size (Kennedy et al. 2004; Lau et al. 2007; Loveless et al. 2006). Proxisome proliferation and the resulting activation of a nuclear receptor [peroxisome proliferator-activated receptor α (PPARα)] have also been proposed as a mechanism for tumor induction and for the immune and hormonal changes seen in rodents (Lau et al. 2007). However, it is not known if this mechanism is relevant to humans, where peroxisome proliferation is generally less apparent (Dewitt et al. 2008; Klaunig et al. 2003). Furthermore, new observations question whether this mechanism is specifically relevant to liver carcinogenesis in rodents (Guyton et al. 2009). This issue remains a subject of active investigation.

In the United States, the only populations exposed above background levels are occupational cohorts and two communities in Minnesota and West Virginia/Ohio. The two communities have been exposed via water contamination coming from adjacent industrial plants; in Minnesota the mean PFOA was 15 ng/mL as measured recently (Minnesota Department of Health 2009), whereas in the Mid-Ohio Valley the mean was 82 ng/mL in 2005 (Emmett et al. 2006a; Frisbee et al. 2009; Steenland et al. 2009a). In the Mid-Ohio Valley population, a great deal of health information and clinical chemistries, as well as serum measurements of PFCs, were gathered in a 2005–2006 survey of 69,000 residents of contaminated water districts. Descriptions of these data have been posted on the C8 Health Project web site (C8 Health Project 2009).

PFOA is often correlated in human serum with another PFC, perfluorooctanesulfonic acid (PFOS). PFOS is also present in most human serum in the United States, with a median serum level of 21 ng/mL, although as with PFOA, the sources of this exposure are poorly characterized (Calafat et al. 2007a). Until recently PFOS has been used in the manufacture of Scotchgard (3M, St. Paul, MN) among other products (also until recently Scotchgard could emit PFOA as it degraded). Its half-life has been estimated at 5.4 years (Olsen et al. 2007a). PFOS is also considered an animal carcinogen by U.S. EPA (2005). The epidemiology of PFOS is even more limited than that of PFOA. We do not review epidemiologic studies of PFOS per se here but mention findings for PFOS in studies that consider both PFOA and PFOS, to help interpret the PFOA results.

## Lipids, Uric Acid, Diabetes, and Cardiovascular and Cerebrovascular Disease

### Lipids

A positive association of PFOA with cholesterol was observed in six occupational studies (Costa et al. 2009; Olsen and Zobel 2007; Olsen et al. 2000, 2003; Sakr et al. 2007a, 2007b), three studies of a highly exposed community (Emmett et al. 2006b; Frisbee et al. in press; Steenland et al. 2009b), and one general population study (Nelson et al. 2010). Six of these studies showed a statistically significant association (at the *p* = 0.05 level; all references in this article to statistical significance refer to *p* ≤ 0.05), whereas two occupational studies (Olsen and Zobel 2007; Olsen et al. 2000) and the smaller of the two community studies (Emmett et al. 2006b) did not. All of these studies were cross-sectional except for three: Sakr et al. (2007b) had data on 454 workers with multiple PFOA and cholesterol measurements over an average of 10 years, Costa et al. (2009) had data on 56 workers with such multiple measurements, and Olsen et al. (2003) had data on 174 workers with two measurements. All three of these studies found a significant correlation over time between cholesterol and PFOA levels.

The risk of high cholesterol (≥ 240 mg/dL) also increased in relation to PFOA in some studies. For example, in Steenland et al. (2009b), comparing the highest quartile of PFOA versus the lowest yielded an odds ratio (OR) for high cholesterol of 1.38 [95% confidence interval (CI), 1.28–1.50].

Many studies also showed positive relationships of PFOA with other lipids (i.e., low density lipoprotein cholesterol and triglycerides), with the exception of high density lipoprotein cholesterol, which was consistently not found to be associated with PFOA. Several studies (Frisbee et al. in press; Nelson et al. 2010; Steenland et al. 2009b) also showed associations of lipids with PFOS that were similar in magnitude to those with PFOA. This finding raises the question of whether all PFCs are associated with increased lipids via some common mechanism. PFOA and PFOS are generally correlated in the serum; in two large studies, the correlations were 0.31 (in a population with high-PFOA exposure; Steenland et al. 2009b) and 0.65 (in a general population with background PFOA and PFOS exposures; Nelson et al. 2010). Generally, models including both compounds showed an attenuation of the association for both PFOA and PFOS, without one being clearly dominant. Only one study (Olsen et al. 1999) addressed the effect of PFOS alone, an occupational cross-sectional study that showed a significant positive correlation with cholesterol at one plant but not at another.

Given the cross-sectional nature of most of these studies, it is not clear whether *a*) PFOA increases cholesterol and other lipids; *b*) both PFOA and cholesterol are jointly affected by some other attribute, substance, or mechanism that causes their correlation; or *c*) high lipids might cause increased retention of PFOA in the body (reverse causality). Some evidence against this last hypothesis is provided by Steenland et al. (2009b), where those who had taken statins, associated with a large decrease in cholesterol, had PFOA levels similar to those who had not taken statins. The three longitudinal studies indicating that cholesterol and PFOA were related over time in subjects with repeated measurements (Costa et al. 2009; Olsen et al. 2003; Sakr et al. 2007b) strengthen the case for a causal relationship, although they do not preclude a relationship in which both PFOA and cholesterol jointly covary with some biologic process that is also changing over time.

The strength of the association of PFOA and cholesterol varied considerably by study, making interpretation even more problematic: The lower the range of PFOA that was studied, the greater the change in cholesterol per unit change in PFOA. Thus, the studies of community populations report larger shifts in cholesterol per unit change in PFOA levels than do the occupational studies (which have higher exposures). Table 1 summarizes the changes in cholesterol associated with changes in PFOA. Assuming a linear relationship between the two (not always the case), the slope relating PFOA to cholesterol varied by two to three orders of magnitude. One possibility that might explain some of this discrepancy would be if the slope of an exposure–response relationship was steep at low PFOA levels and then flattened out, as might be the case, for example, if some biological pathways were saturated. There is a suggestion of such flattening in some studies (Frisbee et al. in press; Steenland et al. 2009b). Most studies did not examine the exposure–response curve in detail.

The findings of associations between PFOA and increased cholesterol in humans contradict what would be expected from animal studies, where PFOA decreases, not increases, serum lipids (Lau et al. 2007). However, as also noted, it is not known whether peroxisome proliferation, the mechanism of action involved, is relevant to humans, where it is generally less apparent.

### Uric acid

Uric acid is a natural product of purine metabolism and has both oxidant and antioxidant properties. There is considerable epidemiologic evidence that elevated uric acid is a risk factor for hypertension (Feig et al. 2008; Shankar et al. 2006).

Three cross-sectional studies report positive associations between PFOA and uric acid. Two are occupational studies [Costa et al. 2009 (*n* = 160); Sakr et al. 2007a (*n* = 1,000)], and one is a community study [Steenland et al. 2010 (*n* = 55,000)]. No detailed results are provided in Sakr et al. (2007a). Costa et al. (2009), using a cross-sectional analysis, found mean uric acid levels of 6.29 μg/mL for 34 currently exposed workers, versus 5.73 μg/mL for 34 matched nonexposed workers (*p* = 0.04). In addition, using repeated measures of both PFOA and uric acid over a 7-year period to perform a longitudinal analysis of this group (*n* = 56), Costa et al. (2009) found a significant positive association between uric acid and PFOA; the two varied together over time.

In the cross-sectional study by Steenland et al. (2010), both PFOA and PFOS were significantly associated with uric acid. An increase from the lowest to highest decile of either PFOA or PFOS was associated with an increase of 0.2–0.3 mg/dL uric acid. Risk of abnormally high uric acid (hyperuricemia, defined as > 6.0 mg/dL for women, > 6.8 mg/dL for men) increased modestly with increasing PFOA; the ORs by quintile of PFOA were 1.00, 1.33 (95% CI, 1.24–1.43), 1.35 (95% CI, 1.26–1.45), 1.47 (95% CI, 1.37–1.58), and 1.47 (95% CI, 1.37–1.58; test for trend, *p* < 0.0001). A positive but less pronounced trend was seen for PFOS. Inclusion of both correlated fluorocarbons in the model indicated that PFOA was a more important predictor than was PFOS. Inclusion of albumin in the model, which binds with PFOA, had little effect on these results.

The overall strength of association between PFOA and uric acid is modest in both the Steenland et al. (2010) and Costa et al. (2009) studies. Causality cannot be inferred from these cross-sectional data. There are mechanisms by which reverse causality might be applicable. For example, uric acid and PFOA may share renal transport systems (organic ion transporters 1 and 3) governing excretion of each substance (Eraly et al. 2008; Nakagawa et al. 2008), so it is possible that if the levels of PFOA increase, the secretion of urate is decreased and therefore blood urate levels may secondarily increase. However, whether this shared transporter hypothesis is relevant in humans remains speculative at this point.

### Cardiovascular disease

Given the findings for cholesterol and uric acid noted above, it is reasonable to ask whether PFOA is associated with cardiovascular disease. The data on cardiovascular disease, however, are limited in quantity and quality, consisting primarily of mortality studies among relatively small cohorts of workers.

The mortality of workers (*n* = 6,207) at a DuPont plant using PFOA, who were employed between 1948 and 2002, has been studied by Leonard et al. (2008) and Sakr et al. (2009). Serum PFOA levels among all workers at this plant in 2004 averaged 240 ng/mL, with an average of 490 ng/mL among workers in the PFOA areas (Sakr et al. 2007a). Standardized mortality ratios (SMRs) were calculated using several different referent populations. SMRs for ischemic heart disease (IHD) compared with U.S. and state general populations were < 1.0, as might be expected because of the healthy worker effect. Sakr et al. (2009) conducted dose–response analyses of these same data using a job–exposure matrix (Kreckmann et al. 2009), which used job categories and measured serum levels for 1,000 workers in 2004 to assign all cohort members to four quartiles of cumulative serum PFOA. The mean serum was 231 ng/mL. Categorical dose–response analyses for IHD using different lags (0. 5, 10, 15, 20 years) and different methods of forming quartiles (based on case exposures or entire cohort exposures) showed no significant positive trends at the *p* = 0.05 level, but the analysis using a 10-year lag and quartiles formed based on the PFOA distribution of the entire cohort did suggest a positive trend [risk ratios (RRs) = 1.0, 1.0, 1.4, and 1.6; *p* for trend = 0.06].

Workers at the 3M plant in Minnesota are the other principal occupational cohort exposed to PFOA. The most recent mortality update of this cohort is from Lundin et al. (2009) (*n* = 3,922). Workers were divided into not exposed, probably exposed, and definitely exposed groups based on job category, with ranges of median serum levels in the probably exposed job categories of 300–1,500 ng/mL and in the definitely exposed job categories of 2,600–5,200 ng/mL. Mortality by the three exposure groups showed rate ratios of 1.0, 1.2 [95% confidence interval (CI), 0.9–1.7), and 0.9 (95% CI, 0.4–2.1), with observed deaths of 92, 103, and only 6 in the definitely exposed job categories. Analyses by cumulative weighted years of exposure (weights for unexposed, probably exposed, and definitely exposed groups were 1, 30, and 100) showed rate ratios of 1.0 (138 observed), 1.2 (95% CI, 0.9–1.8; 42 observed), and 0.8 (95% CI, 0.5–1.2; 21 observed). There is little or no evidence of a positive trend in these data. Cumulatively, the two studies provide insufficient data for inferences regarding an association between PFOA and cardiovascular disease.

Finally, Melzer et al. (2010) found no trend in self-reported history of heart disease in data from the National Health and Nutrition Examination Survey (NHANES), after dividing PFOA serum levels into quartiles. These data are limited by the low range of exposure (background levels in the general population), the cross-sectional nature of the data, and the lack of validation of self-reported data.

### Cerebrovascular disease

Cerebrovascular disease is of interest given the findings for uric acid, which is related to blood pressure, a major determinant of cerebrovascular disease. (No studies directly address PFOA and blood pressure.) Again, however, the epidemiology is limited to the two principal occupational studies described above. Leonard et al. (2008) found a deficit of cerebrovascular mortality in comparing exposed DuPont workers with nonexposed DuPont workers (SMR = 0.86; 95% CI, 0.60–1.20). Lundin et al. (2009) found a suggestion of a positive trend of stroke mortality across nonexposed, probably exposed, and definitely exposed job categories using SMRs [SMRs of 0.5 (95% CI, 0.3– 0.8), 0.7 (0.4–1.1), and 1.6 (0.5–3.7), respectively; no test for trend presented]. Using a cumulative measure of weighted years of exposure, the rate ratios were 1.0 (23 deaths), 0.6 (95% CI, 0.2–2.2; 3 deaths), and 2.1 (95% CI, 1.0–4.6; 9 deaths), for < 1 year, 1–5 years, and > 5 years, respectively. Like the data for heart disease, these data are too sparse to draw any conclusions.

### Diabetes

Diabetes is not a disease of particular interest based on animal data, but some epidemiologic studies have addressed this end point nonetheless. Leonard et al. (2008) found a deficit of diabetes mortality in comparing exposed DuPont workers with the U.S. population but a 2-fold increase in diabetes mortality compared with other, nonexposed DuPont workers (SMR = 1.97; 95% CI, 1.23–2.98; 22 deaths), arguably a more suitable comparison group because of the healthy worker effect. Similarly, in internal comparisons among 3M workers, Lundin et al. (2009) found an excess of diabetes mortality in comparing a combined category of probably and definitely exposed workers and nonexposed workers (RR = 3.7; 95% CI, 1.4–10.1; 18 vs. 5 deaths). This finding was much less pronounced using years of exposure weighted by a qualitative judgment of intensity.

Diabetes mortality is not the optimal measure for a largely nonfatal disease. MacNeil et al. (2009) studied diabetes prevalence in a community exposed to high levels of PFOA via contaminated drinking water near a DuPont plant. Self-reported diabetes prevalence (7.8%) and date of onset were collected from 54,468 adults, who had a median serum PFOA level of 28 ng/mL (mean = 87 ng/mL) in 2005–2006 at the time of the survey. Overall, there was no relation between diabetes prevalence, either self-reported or validated by medical records, and serum PFOA as measured at the time of the survey. A nested case–control analysis was conducted in which the population was restricted to participants who had at least 20 years of continuous residence in a specific water district (*n* = 13,922), to maximize the likelihood that serum levels in 2005–2006 would reflect the relative exposure of subjects in the past. Cases were restricted to those validated by medical records and occurring in the 10 years before 2005 (*n* = 1,055). There was a negative trend in diabetes occurrence by increasing serum PFOA decile (ORs = 1.00, 0.71, 0.60, 0.72, 0.65, 0.65, 0.87, 0.58, 0.62, 0.72). In additional analyses, fasting serum glucose showed no consistent trend by increasing decile of serum PFOA.

### Cancer

Animal data suggest that pancreatic, testicular, liver, and perhaps breast cancer may be related to PFOA exposure (Lau et al. 2007). Human studies are again restricted to the two U.S. occupational cohorts, as well as one follow-up study of the general population in Denmark. Leonard et al. (2008) found no excesses for liver, pancreas, testicular, or breast cancer based on small numbers of deaths (8, 11, 1, and 2 respectively). A 2-fold excess of kidney cancer was observed (SMR = 1.81; 95% CI, 0.94–3.16; 12 deaths). Lundin et al. (2009) similarly found no excesses of liver and pancreatic cancers based on small numbers (3 and 13 deaths, respectively) and did not report on testicular or kidney cancer. Internal analyses by job category were reported for three cancers (prostate, pancreas, bladder). Some positive trend for prostate cancer was found by exposure group based on job category [nonexposed, probably exposed, definitely exposed; RRs of 1.0, 3.0 (95% CI, 0.9–9.7), and 6.6 (95% CI, 1.1–37.7), based on 4, 10, and 2 cases respectively. For pancreatic cancer, the corresponding RRs were 1.0 and 1.7 (95% CI, 0.5–4.8), respectively, for nonexposed and for combined probable/definite exposure groups. The data from both occupational cohorts are clearly quite sparse and prohibit any firm conclusions.

There is also a study of 55,053 Danish adults 50–65 years of age who were followed from enrollment in 1993–1997 until 2006 through linkage with the Danish cancer registry (Eriksen et al. 2009). There were 713, 332, 128, and 67 incident cases of prostate, bladder, pancreatic, and liver cancers found in this period. A case–cohort approach was used. Dividing the population into quartiles of serum PFOA (based on the cases), no significant linear trends by quartile were seen for any of the four cancers studied, although modest positive associations with prostate and pancreas cancers were reported [RR for highest quartile vs. lowest quartile: prostate cancer, 1.18 (95% CI, 0.84–1.65); pancreatic cancer, 1.55 (95% CI, 0.85–2.80)]. Trends were of borderline significance for prostate cancer and pancreatic cancer and were absent or negative for bladder and liver cancer. Findings for PFOS largely paralleled those for PFOA, which is not surprising given that the Spearman correlation between the two was 0.80. This study, although much larger than the occupational studies cited above, had much lower ranges of PFOA exposure (mean = 6–7 ng/mL), typical of a general population.

## Immune, Thyroid, Liver, and Kidney Function and Sex Hormones

### Immune function

Animal studies have demonstrated that PFOA causes lymphoid organ atrophy and decreased *de novo* antibody production in certain strains of mice (DeWitt et al. 2008; Yang et al. 2000). Total doses received in the Dewitt et al. (2008) study appear to have been about an order of magnitude higher than the total lifetime dose received by people drinking water contaminated with 1 ng/mL PFOA. There appears to be strain as well as species sensitivity to the immunotoxic effects of PFOA, with rats being considerably less sensitive than mice (Loveless et al. 2008), leading some to question the relevance of these data to humans. Immunotoxicity by PFOA in rodents appears to occur both through and independent of peroxisome proliferation (Yang et al. 2000). Immunosuppression, at least in part, occurs in exposed mice lacking the PPARα receptor (DeWitt et al. 2009). There is also evidence from animal studies that PFOA can suppress inflammatory responses (Taylor et al. 2006), which is similar to the effects of other PPAR agonists (DeWitt et al. 2009).

Human studies to date are very limited and have included only relatively insensitive test measures, such as white blood cell count or serum immunoglobulin (Ig) levels. Emmett et al. (2006a) examined the effects on PFOA on hematologic parameters in a cross-sectional community study composed of residents who lived in a contaminated water district for at least 2 years and a smaller group of volunteers who met the same eligibility criteria (Emmett et al. 2006b). They found no association between serum PFOA and lymphocytes, neutrophils, eosinophils, or basophils. There was one small statistically significant association between PFOA and absolute monocyte counts; elevated numbers of monocytes are often associated with recent infection, which was not controlled in the analysis.

Costa et al. (2009) measured serum levels of IgG, IgM, and IgA in 34 male workers engaged in the production of PFOA. Almost 13% of the workers showed levels of serum IgA outside the laboratory referent values. Because of the small population and lack of controls, it is difficult to draw any conclusions from these data. Furthermore, the levels of serum Igs, which represent circulating antibodies, are extremely variable in the population and relatively insensitive markers for immune system effects.

Overall human studies are insufficient for assessing whether PFOA is or is not related to immune suppression.

### Thyroid function

After experimental evidence of PFCs reducing T_3_ and T_4_ in rats and monkeys (Butenhoff et al. 2002; Lau et al. 2007), a number of occupational and community studies have investigated these clinical markers of thyroid function, usually in cross-sectional studies. Emmett et al. (2006b) investigated the association between PFOA and health outcomes in 371 community residents with high serum levels (median, 534 ng/mL) due to contaminated drinking water. No significant association was found between TSH and the serum level of PFOA. In a small community study (*n* = 31) of New York State anglers, at much lower exposure levels (PFOA serum geometric mean = 1.33 ng/mL), potential associations were investigated between serum concentrations of eight measured PFCs and levels of TSH and free T_4_ (Bloom et al. 2010). No associations were found, but study power was very low.

Olsen et al. (2003) conducted a cross-sectional analysis that included two plants with 255 and 263 workers, respectively. Multivariate regression analysis by quartiles of PFOA or PFOS exposure showed no significant association of either compound with T_3_, T_4_, or TSH. A continuous regression suggested a slight positive association between log PFOA and log T_3_ (coefficient = 0.016, *p*-value = 0.01); however, the contribution of this association to the variance of the outcome was negligible (partial *R*^2^ = 0.01). A longitudinal analysis of up to three examinations for 174 workers from the same company found no association between PFOA and thyroid hormones (Olsen et al. 2003).

In a more recent occupational study, Olsen and Zobel (2007) reported on analyses of thyroid hormones in relation to PFOA among 552 employees in three plants (median serum level, 1,100 ng/mL). A log–log regression adjusted for age, body mass index (BMI), and alcohol intake showed a negative slope for free T_4_ (*p* = 0.01) and positive slope for T_3_ (*p* = 0.05), with consistent direction of effect for each plant. For total T_4_ and TSH, correlations across plants and within plants were not significant.

A recent cross-sectional analysis of self-reported thyroid disease in the NHANES in relation to either PFOA or PFOS reported a significant association for PFOA in females, and a similar but much less precise association in males, but no association for PFOS (Melzer et al. 2010). The OR for females of ever having had thyroid disease for the upper quartile of PFOA serum levels was 1.64 (95% CI, 1.09–2.46) compared with the first quartile, and for current thyroid disease plus thyroid hormone medication the equivalent OR was 1.86 (95% CI, 1.12–3.09). For men the respective findings were 1.58 (95% CI, 0.74–3.39) and 1.89 (95% CI, 0.60–5.90). These findings are striking given the very low exposures compared with the occupational studies, but the study suffers from using self-reported diagnoses, having no thyroid hormone data, having no exposure data at the time of diagnosis, and not distinguishing between hypo- and hyperthyroidism. In addition, there is some concern that the disease or treatment of disease may affect serum levels of PFOA.

Overall, the occupational data at high exposure levels show no consistent evidence of a reduction of thyroid hormone. In addition associations between T_3_ and T_4_ measured by the more common analog methods need to be treated with some caution because free T_4_ assay results have been reported to be affected by the presence of PFOS, which can lead to apparent positive associations (Chang et al. 2007). It is not known whether this is a problem with PFOA as well. Two community studies at lower exposure levels did not indicate an effect, but study sizes were small. The NHANES data show a strong effect on self-reported thyroid disease at very low levels, not easily interpreted in the context of the other studies.

### Sex hormones

Increases in estradiol and decreases in testosterone with PFOA exposure have been observed in rodents (Lau et al. 2007). However, there are very limited human data on hormones in relation to serum PFOA, found in three occupational cross-sectional studies. Olsen et al. (1998) studied 191 male workers divided into four exposure groups and measured estradiol, 17-hydroxyprogesterone, prolactin, and bound testosterone. No significant associations between exposure and these hormones were found, but most of the population were in the two lowest exposure groups (< 1 and < 10 ng/mL), with only five subjects in the > 30 group. Costa et al. (2009) reported a multivariate regression of estradiol and testosterone in relation to PFOA in a group of 56 workers followed in routine occupational surveillance, in which there were no associations of sex hormones with serum PFOA. Finally, Sakr et al. (2007a) found a significant association between serum PFOA and both estradiol and testosterone in men (*n* = 782) in linear regression models (regression coefficients = 22.3 and 0.6, respectively; *p*-values = 0.017 and 0.034, respectively). Apparently there were no significant findings for hormones in the 243 women.

### Liver function

Studies of the distribution of PFOA in rodents have found relatively high concentrations in the liver. Increased liver weight has been found in rodents and nonhuman primates exposed experimentally to PFOA (Lau et. at 2007). In rodents, but not necessarily in primates, liver toxicity is related to the PPARα mode of action. However, the nonhuman primate studies and the evidence of toxicity in PPAR-null mice suggest that liver toxicity via other modes of action may be relevant, including other nuclear receptors in the liver (Dewitt et al. 2009; Ren et al. 2009). The animal findings on liver toxicity have prompted a number of studies of liver enzymes in people exposed to PFOA.

Emmett et al. (2006b) reported on the association between serum levels and several liver enzymes in serum among 371 community residents who were exposed to PFOA (median exposure, 354 ng/mL). Enzymes included alkaline phosphatase (ALP), aspartate aminotransferase (AST), alanine aminotransferase (also known as serum glutamic pyruvic transaminase), and gamma glutamyl transpeptidase (GGT). No correlations of serum PFOA and enzymes approached statistical significance. One of the comparisons for abnormal values (AST; abnormal not defined) was associated with PFOA, but the normal group had higher PFOA, suggesting a protective rather than a harmful effect of PFOA.

Costa et al. (2009) reported on a surveillance program of workers in a PFOA plant. Bilirubin and four liver enzymes showed no associations in cross-sectional analyses, but in a longitudinal analysis (*n* = 56) all four enzymes indicated a positive slope of increasing enzyme levels with respect to PFOA, three with *p*-values < 0.05 [alanine transaminase (ALT), GGT, and ALP] and one (AST) not significant. Results are presented in terms of regression coefficients for log-transformed variables, which are sometimes hard to interpret in terms of strength of effect. Total bilirubin fell with increasing PFOA (*p* < 0.01).

Sakr et al. (2007b), in a longitudinal study of liver enzymes in an occupational population (*n* = 454; mean serum PFOA = 1,130 ng/mL), found significant associations with AST (0.35 units increase per 1,000 ng/mL PFOA; 95% CI, 0.10–0.60). Total bilirubin declined with increasing PFOA (0.008 mg/L decline per 1,000 ng/mL; 95% CI, −0.0139 to −0.0021). In each of these cases, the change was small compared with the mean and standard deviation of these clinical markers.

Sakr et al. (2007a) also conducted a cross-sectional study at the same plant of PFOA, lipids, and liver enzymes, in 1,025 workers with potential exposure to PFOA. AST, ALT, and total bilirubin were not significantly associated with the level of PFOA, but GGT was significantly positively associated with PFOA (*p* = 0.02). Observed increases were no longer significant when the analysis was restricted to those not on lipid-lowering medications. No notable change was found for the log of total bilirubin (Sakr et al. 2007a).

Several reports by Olsen and colleagues have addressed the liver enzymes AST, GGT, and ALP along with bilirubin in exposed workers; in the most recent and extensive analysis, Olsen and Zobel (2007) reported data from 552 volunteers from three plants (mean serum level = 2,210 ng/mL). For the three plants together, there was a modest positive association for log GGT (coefficient = 0.0326, *p* = 0.05) and log ALT (coefficient = 0.0249, *p* = 0.06), in a model with BMI but not in a model with triglycerides, but no evidence of an effect for ALP or AST in either model. However, results were inconsistent across plants, with these overall positive results derived from just of one of the four plants included. For bilirubin, the relationship was consistently negative and significantly negative overall (exposure–response coefficient: in model with BMI, −0.0325, *p* = 0.001; in model with triglycerides, −0.0267, *p* = 0.01). These positive findings contrast with earlier cross-sectional analyses of these plants that were essentially negative (e.g., Gilliland and Mandel 1996; Olsen et al. 1999).

Lin et al. (2010) assessed the relationships between ALT, GGT, and bilirubin and four PFCs, including PFOS and PFOA, measured in serum in the NHANES surveys of 1999–2000 and 2003–2004 (*n* = 2,216). They found significant trends for liver enzymes across quartiles of PFOA and in regressions of log-transformed enzyme levels against log-transformed PFOA. However exposure contrasts were low, with the lowest quartile < 2.9 and the top quartile > 5.95 ng/mL PFOA. Given these small exposure contrasts, the models suggested a much larger effect per unit exposure than did other studies.

In summary, studies of liver enzymes have reported some associations with PFOA. Four independent studies (two longitudinal and two cross-sectional studies) reported reduced bilirubin associated with increasing PFOA, and at least two studies showed results suggesting increasing AST or GGT in relation to PFOA. However, the changes in liver enzymes are quite small, and the magnitude of effect is quite inconsistent, with results from the NHANES study showing an effect of exposure going from 1 to 3 ng/mL, whereas other studies looked at contrasts of thousands of nanograms per milliliter of PFOA, in some cases with no apparent association. These results provide limited evidence of some biological activity of PFOA in human liver, the clinical significance of which is uncertain.

### Kidney function

PFOA concentrates in the kidney in animals and therefore is of interest as a potential target organ. However, no animal data document kidney toxicity, and human data are also quite limited.

In an occupational study, Costa et al. (2009) reported no significant association between PFOA and either urea nitrogen or creatinine. These negative results were consistent with those of Emmett et al. (2006b), who found no significant association between either blood urea nitrogen or creatinine with the serum level of PFOA.

## Reproductive and Developmental Outcomes

Toxicology studies indicate the potential for PFOA and PFOS to affect fetal growth and development. *In utero* exposure to PFCs is associated with a range of nonspecific adverse developmental outcomes in mouse, rat, and rabbit models (Lau et al. 2007; Olsen et al. 2009), including reduced fetal weight and increased neonatal mortality. Extrapolation across species and end points is particularly problematic in reproductive toxicology.

Epidemiologic research on PFOA and reproductive end points was initiated quite recently, with most studies published in 2007 or later (Olsen et al. 2009). With few exceptions, the studies examine serum PFOA in relation to the risks of a range of different adverse outcomes. Except for fetal growth, most of the end points have been examined in only one or two studies.

Starting with fertility, one study of semen quality and reproductive hormones in men (Joensen et al. 2009) reported decrements in sperm count and number of morphologically normal sperm with higher exposure to the combined level of PFOA and PFOS, but weaker associations with PFOA alone. An integrated measure of couple fecundity, time to pregnancy, was examined by Fei et al. (2009). This same population was examined for several other reproductive end points and thus warrants a more extensive description.

Starting with > 100,000 participants in the Danish National Birth Cohort, which recruited women from 1996 to 2002 and followed women through the course of pregnancy and continued with childhood assessment, Fei et al. (2009) randomly selected 1,400 women with singleton live births for measurement of PFOA and PFOS in blood collected during the first trimester of pregnancy (mean PFOA = 5.6 ng/mL). The evaluation of time to pregnancy showed increased risk of irregular menstrual cycles in the upper three quartiles of PFOA relative to the lowest quartile (15.0% vs. 9.0%) and an increase in mean PFOA with increasing time required to conceive (5.4 ng/mL for < 6 months to conception, 6.0 ng/mL for 6–12 months to conception, and 6.3 ng/mL for > 12 months to conception). The odds of infertility (≥ 12 months without conception) were elevated in the upper three quartiles of PFOA exposure (ORs ranging from 1.6 to 2.5) relative to the lowest quartile. Very similar patterns were reported for PFOS. The absence of dose–response gradients for fertility across levels suggests the possibility of some peculiarity in the lowest exposure group but does not preclude a causal relationship with a maximum effect at a very low exposure level.

The course of pregnancy, including risk of miscarriage and preeclampsia, has been addressed in a study of a subset of women exposed to markedly elevated levels of PFOA in the Mid-Ohio Valley (Stein et al. 2009). No association was found between PFOA and miscarriage, whereas a weak association was found for preeclampsia (for above-median exposure to PFOA: OR = 1.3; 95% CI, 0.9–1.9). Nolan et al. (2009a) used birth certificate information to address pregnancy complications in women residing in a high-PFOA area of the Mid-Ohio Valley and reported extremely imprecise associations with anemia and dysfunctional labor and lower overall risk of labor and delivery complications.

The most extensive set of studies has examined fetal growth, birth weight, duration of gestation, and related indices of *in utero* development. Seven studies reported continuous measures of birth weight in relation to continuous measures of PFOA exposure (Apelberg et al. 2007; Fei et al. 2007; Hamm et al. 2009; Inoue et al. 2004; Monroy et al. 2008; So et al. 2006; Washino et al. 2009). Two studies of background exposure to PFOA reported clear evidence of decreased birth weight in relation to increased PFOA (Apelberg et al. 2007; Fei et al. 2007), with smaller decrements reported in two other studies of background exposure levels (Hamm et al. 2009; Washino et al. 2009). Magnitude of association ranged from a decrement of 37 to 104 g per log unit increase in PFOA exposure, with varying degrees of statistical precision, with one study (Fei et al. 2007) reporting a smaller reduction of 10.6 g per ng/mL PFOA. Three smaller studies reported no association between PFOA and birth weight (Inoue et al. 2004; Monroy et al. 2008; So et al. 2006). Risk of low birth weight (< 2,500 g) was elevated in the upper three quartiles relative to the first quartile in the analysis of the Danish National Birth Cohort (Fei et al. 2007), but a measure of small-for-gestational-age (which accounts for duration of gestation) was not related to PFOA in the Danish National Birth Cohort (Fei et al. 2007) nor in a study from Alberta, Canada (Hamm et al. 2009). An ecologic study of residence and birth weight in the Mid-Ohio Valley found no association between PFOA exposure and birth weight (Nolan et al. 2009b). Stein et al. (2009) did not find an association between PFOA and self-reported low birth weight.

Cumulatively, the studies provide inconsistent suggestions of a possible decrement in birth weight associated with PFOA exposure, with studies varying in whether the association with PFOS is similar (Apelberg et al. 2007), stronger (Stein et al. 2009; Washino et al. 2009), or weaker (Fei et al. 2009; Hamm et al. 2009) than that reported for PFOA. Different measures of fetal growth across studies make it difficult to assess whether the studies of background PFOA and those addressing more elevated PFOA levels are consistent with one another, but thus far the positive evidence for an effect on birth weight comes from studies of background exposure levels. Although an adverse impact of PFOA on birth weight would be of some concern in suggesting sensitivity of the fetus, shifts of the reported magnitudes within the normal birth weight range are of little or no clinical significance.

Other indices of fetal growth have been examined in isolated studies with suggestions of adverse effects of PFOA on head circumference and ponderal index (Apelberg et al. 2007) and on abdominal circumference and birth length (Fei et al. 2008b). Studies addressing preterm birth reported elevated risks for intermediate exposure categories only (Fei et al. 2007) or no association (Hamm et al. 2009; Stein et al. 2009).

Child health and development have been examined to a very limited extent. Stein et al. (2009) reported an association between PFOA levels exceeding the 90th percentile and birth defects in aggregate (OR = 1.7; 95% CI, = 0.8–3.6). Birth defects in aggregate were not associated with residence in a high-PFOA area of the Mid-Ohio Valley in a study based on birth certificates (Nolan et al. 2009a). Fei et al. (2008a) examined parent-reported developmental milestones in infants 6 and 18 months of age and found no indication of delays associated with higher PFOA exposure.

## Discussion

Although the epidemiologic literature on the health effects of PFOA is growing rapidly, overall it remains limited in volume and quality. Many studies are cross-sectional in nature, making causal inference difficult. Other studies are small and have too few outcome events to draw firm conclusions. The range of PFOA varies greatly in different populations studied, with some studies of biomarkers identifying effects at low general population levels and others with a much higher and large range of exposures finding smaller or no effects.

The most consistent findings have been for modest increases in cholesterol, and to a lesser degree for a modest increase in uric acid, among those with higher PFOA levels. However, these findings have been largely based on cross-sectional data (exposure and outcome measured simultaneously), calling into question whether the relationship is causal. Three longitudinal studies show that PFOA changes over time correlate with changes in cholesterol levels (Costa et al. 2009; Olsen et al. 2003; Sakr et al. 2007b), and one longitudinal study shows a similar effect on uric acid (Costa et al. 2009). These longitudinal studies strengthen the case for a causal relationship, although they do not necessarily preclude that PFOA and cholesterol, or PFOA and uric acid, are both associated with some other biological process that is changing over time. Other cross-sectional findings show some evidence of an increase in liver enzymes with higher serum PFOA, but these findings have not been consistent across studies, and observed enzyme increases have been modest.

Cohorts followed for mortality have the potential to shed more light on the occurrence of chronic disease. There have been only two of these, both occupational (Leonard et al. 2008; Lundin et al. 2009). Findings in these two cohorts have not been consistent, with the exception of diabetes mortality, which was significantly elevated in both studies when worker comparison groups were used. However, a large study of diabetes prevalence, a more sensitive outcome, using retrospective exposure estimates, showed no relation with PFOA (MacNeil et al. 2009). Other findings from the occupational cohort mortality studies have been inconsistent, with excesses in one but not the other. There is some evidence of increased kidney cancer and heart disease in one cohort (Leonard et al. 2008; Sakr et al. 2009), whereas stroke and prostate cancer showed a positive exposure–response trend in the other (Lundin et al. 2009). All these findings (except for the heart disease finding) were based on small numbers of deaths. Another nonoccupational cohort, with much lower exposure levels, has been followed for cancer in Denmark, with no significant excesses observed (Eriksen et al. 2009). In summary then, for chronic disease, sparse and inconsistent evidence prohibits any conclusions.

The research on reproductive and developmental health end points is expanding rapidly and offers sporadic evidence that is difficult to interpret. A range of indicators of fetal development, including birth weight, may be subtly affected by relatively low levels of PFOA, but the potential for an artifactual relationship with PFOA metabolism cannot be discounted (Savitz 2007). Furthermore, the associations, although often statistically significant, are quite small in absolute terms and of limited direct clinical significance. Other studies that address more clinically consequential outcomes report null or marginal adverse effects, with none replicated in independent studies at this time.

In summary, the epidemiologic evidence accumulated thus far on health effects of PFOA comes principally from two occupational cohorts and several occupational cross-sectional studies, as well as community populations with background exposure levels, and one community with elevated exposure in the Mid-Ohio Valley.

Although the occupational studies have the advantage of substantially elevated exposure ranges, they suffer from several limitations. They are primarily limited to males. The cohort mortality data are limited for rare diseases and those that are typically not fatal. The prevalence studies measure subclinical end points, typically blood chemistry, and are limited for causal inference and are of variable clinical significance.

The community studies of populations with background exposures are more broadly representative of the general population, but it is difficult to discern clear associations within the limited exposure range available to study. In contrast to these important but inherently limited sources of epidemiologic information, we are pursuing an unusual opportunity to extend our knowledge regarding the health effects of PFOA considerably by conducting a series of studies of an exposed community population in the Mid-Ohio Valley (Frisbee et al. 2009) (see http://www.c8sciencepanel.org for a description of the studies currently under way). Levels of PFOA exposure in this population are markedly above background (median values were 5 times background but included a wide range; mean levels were 15 times background), although still below occupational levels (which often have mean levels 200 times background). The population is large (> 60,000 individuals) and comprises a wide age range (including children). In addition, we are also studying disease incidence in a cohort of approximately 6,000 workers with much higher levels (e.g., levels of 1,000 ng/mL, 200 times background). [Leonard et al. (2008) studied this same cohort for mortality.] Although there are methodologic challenges in this setting as well, particularly in reconstructing historical exposure levels and in ascertaining health end points accurately, these studies should provide new evidence complementary to that which has been generated to this point. To the extent that there are other such communities around the world that are exposed to elevated levels of PFOA due to a point source, epidemiologic research in those settings would be of clear value and help to evaluate the consistency of future findings in the Mid-Ohio Valley.

## Figures and Tables

**Table 1:** Changes in cholesterol in relation to changes in PFOA levels.

	Study description	Change in PFOA (ng/mL)	Change in cholesterol (mg/dL)	Slope (assumes linearity)^a^
Frisbee et al. in press	Cross-sectional; 12,476 high-exposed children; mean PFOA = 69 ng/mL	400	10	0.03
Steenland et al. 2009b	Cross-sectional; 46,294 high-exposed adults; mean PFOA = 80 ng/mL	340	11	0.03
Sakr et al. 2007a	Cross-sectional; 1,024 workers; mean PFOA = 428 ng/mL	1,000	5	0.005
Sakr et al. 2009b	Longitudinal; 454 workers; mean PFOA = 1,130 ng/mL	1,000	1	0.001
Nelson et al. 2010	Cross-sectional; 860 adults, general population; mean PFOA = 4 ng/mL	5	10	2.0
Costa et al. 2009	Longitudinal, 54 workers; mean PFOA ~ 12,000 ng/mL^b^	NA	NA	0.001
Emmett et al. 2006b	Cross-sectional; 371 high-exposed adults; median PFOA, 354 ng/mL	4,000 (estimated from slope)	22	0.006^c^
Olsen et al. 2000	Cross-sectional (three time points); 111, 80, and 74 workers; mean PFOA ~ 22,000 ng/mL	~ 22,000	~ 16	0.0007^c^
Olsen and Zobel 2007	Cross-sectional, 506 workers; mean PFOA = 2,210 ng/mL^d^	NA	NA	0.001
Olsen et al. 2003	Longitudinal, 174 workers; mean PFOA ~ 1,500 ng/mL^b^	NA	NA	0.001

NA, not available.

a Change in cholesterol per ng/mL change in PFOA, assuming a linear relationship, which is not always apparent in some studies. Slopes were calculated for this table from published data; some studies presented different results for different subsamples.

b Results were presented for the log of cholesterol versus parts per million PFOA (coefficients = 0.032 and 0.028, respectively), prohibiting extraction of a linear slope.

c Not significant at *p* = 0.05.

d The result presented was the coefficient relating the log of cholesterol to the log of PFOA in parts per million (0.0076). We have approximated a linear slope for the range 0–1,000 ng/mL by using predicted cholesterol values at those two points.
